# Frequency, risk factors and feto-maternal outcomes of abruptio placentae in Northern Tanzania: a registry-based retrospective cohort study

**DOI:** 10.1186/s12884-015-0678-x

**Published:** 2015-10-07

**Authors:** Godwin S. Macheku, Rune Nathaniel Philemon, Olola Oneko, Pendo S. Mlay, Gileard Masenga, Joseph Obure, Michael Johnson Mahande

**Affiliations:** Department of Obstetrics and Gynaecology, Kilimanjaro Christian Medical Centre (KCMC)/Kilimanjaro Christian Medical Collage (KCM College), Moshi, Tanzania; Department of Epidemiology & Biostatistics, Institute of Public Health, Kilimanjaro Christian Medical University College, 2240, Moshi, Tanzania; Department of Paediatrics, Kilimanjaro Christian Medical Centre (KCMC)/Kilimanjaro Christian Medical Collage (KCM College), Moshi, Tanzania

**Keywords:** Frequency, Abruptio placentae, Risk factors, Feto-maternal outcomes

## Abstract

**Background:**

Abruptio placentae remains a major cause of maternal and perinatal morbidity and mortality in developing countries. Little is known about the burden of abruptio placentae in Tanzania. This study aimed to determine frequency, risk factors for abruptio placentae and subsequent feto-maternal outcomes in women with abruptio placentae.

**Methods:**

We designed a retrospective cohort study using maternally-linked data from Kilimanjaro Christian Medical Centre (KCMC) medical birth registry. Data on all women who delivered live infants and stillbirths at 28 or more weeks of gestation at KCMC hospital from July 2000 to December 2010 (*n* = 39,993) were analysed. Multivariate logistic models were used to calculate odds ratios (OR) and 95 % confidence intervals (CIs) for risk factors, and feto-maternal outcomes associated with abruptio placentae.

**Results:**

The frequency of abruptio placentae was 0.3 % (112/39,993). Risk factors for abruptio placentae were chronic hypertension (OR 4.1; 95 % CI 1.3–12.8), preeclampsia/eclampsia (OR 2.1; 95 % CI 1.1–4.1), previous caesarean delivery (OR 1.3; 95 % CI 1.2–4.2), previous abruptio placentae (OR 2.3; 95 % CI 1.8–3.4), fewer antenatal care visits (OR 1.3; 95 % 1.1–2.4) and high parity (OR 1.4; 95 % CI 1.2–8.6). Maternal complications associated with abruptio placentae were antepartum haemorrhage (OR 11.5; 95 % CI 6.3–21.2), postpartum haemorrhage (OR 17.9; 95 % 8.8–36.4),), caesarean delivery (OR 5.6; 95 % CI 3.6–8.8), need for blood transfusions (OR 9.6; 95 % CI 6.5–14.1), altered liver function (OR 5.3; 95 % CI 1.3–21.6) and maternal death (OR 1.6; 95 % CI 1.5–1.8). In addition, women with abruptio placentae had prolonged duration of hospital stay (more than 4 days) and were more likely to have been referred during labour. Adverse fetal outcomes associated with abruptio placentae include low birth weight (OR 5.9; 95 % CI 3.9–8.7), perinatal death (OR 17.6; 95 % CI 11.3–27.3) and low Apgar score (below 7) at 1 and 5 min.

**Conclusions:**

Frequency of abruptio placentae is comparable with local and international studies. Chronic hypertension, preeclampsia, prior caesarean section delivery, prior abruptio placentae, poor attendance to antenatal care and high parity were independently associated with abruptio placentae. Abruptio placentae was associated with adverse maternal and foetal outcomes. Clinicians should identify risk factors for abruptio placentae during prenatal care when managing pregnant women to prevent adverse maternal and foetal outcomes.

## Background

Abruptio placentae is defined as placental detachment before and during delivery [1]. It occurs in about 0.38–1 % of singleton births, and the incidence increases among twin pregnancies, ranging from 1 to 2 % [2, 3]. It is a major obstetric complication associated with an increased risk of foetal and maternal morbidity and mortality globally, especially in developing countries [1, 4–6]. Over 50 % of all perinatal deaths attributed to abruptio placentae pregnancies are accompanied by premature delivery [7]. In addition, abruptio placentae accounts for 20–25 % of antepartum haemorrhages, and it is also associated with an increased risk of disseminated intravascular coagulopathy, severe maternal shock, renal failure [1, 8], postpartum haemorrhage and maternal death [1, 9, 10]. Furthermore, abruptio placentae has been associated with adverse foetal outcomes including low birth weight, preterm birth, intrauterine growth restriction, birth asphyxia, fetal distress, low apgar score, transfer to neonatal intensive care unit, stillbirth [1, 10–12], congenital anomalies and perinatal death ranging from 4.4 to 67.3 % [1, 9, 13].

Several risk factors have been associated with abruptio placentae including previous history of small-for gestational age delivery, young or advanced maternal age (<20 years or >=35 years), previous history of abruptio placentae, previous history of caesarean section, maternal headaches/migraine, current diabetes mellitus, multiparity (especially grand multiparity), maternal cigarette smoking, multiple gestations, chronic hypertension, preeclampsia, premature rupture of membranes, thrombophilic disorders, abdominal trauma and polyhydramnios [4, 9, 14–21].

Most studies on abruptio placenta have been reported in developed countries, there are limited reports from Tanzania and the East African region. Given the high background risk of African women coupled with weak health care system, some women in Africa might carry an exceptionally high risk of abruptio placentae as compared to women in developed countries and may benefit from preventive interventions thus the need to identify those at risk. The objective of this study was to determine the frequency, risk factors for abruptio placentae and feto-maternal outcomes associated with abruptio placentae in Tanzania.

## Methods

### Study setting

This study was conducted at Kilimanjaro Christian Medical Centre (KCMC) in Northern Tanzania. KCMC is one among the four zonal referral hospitals in Tanzania. It is located in Moshi Municipality in Northern Tanzania. The catchment area of the hospital captures the regions Kilimanjaro, Arusha, Manyara and Tanga, although most deliveries it taking place at the hospital are from the Kilimanjaro region. Annually, around 4000 deliveries are conducted at KCMC. The KCMC medical birth registry was established in 1999 as a collaborative project between KCMC through Kilimanjaro Christian Medical University College and the medical birth registry of Norway through University of Bergen, and it has been in operation since 2000. From this period, information regarding all deliveries that occur in the department of Obstetrics and Gynaecology at KCMC is collected.

### Study design

**Fig. 1:**
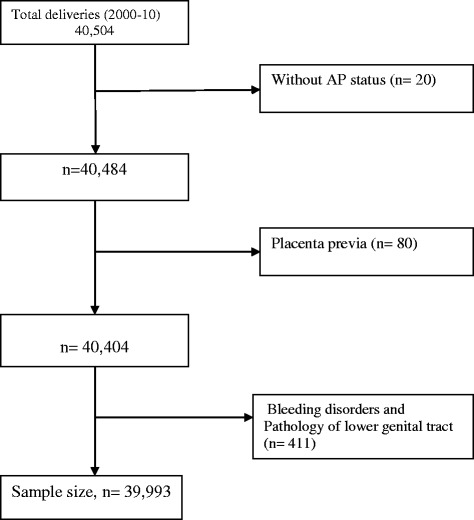
Schematic diagram for selected study participants

A retrospective cohort study was designed using maternally-linked data from KCMC-medical birth registry. A unique hospital identification number which was assigned to each woman at first admission was used to trace her medical records for later admissions. This identification number was included in the registry’s record of each birth and was used to link records of successive births of the same woman. This enabled us to create historic cohort of women who delivered at the hospital from July, 2000 to December, 2010 (*n* = 40, 504). All women with 28 or more weeks of gestation were included. We excluded all patients diagnosed with placentae previa, pathology of lower genital tract, patients who had bleeding disorders (i.e. maternal bleeding disorders unrelated to the effect of abruptio placentae) and those with missing record on abruptio placentae status (*n* = 511). The remaining 39,993 births (112 abruptio placentae case and 39,881 non-abruptio placentae) were analysed (Fig. 1).

### Data collection

Information for all women who delivered at Obstetrics and Gynaecology unit was recorded at the medical birth registry in a computerized data base. Trained midwives conducted an interview for all mothers who delivered on daily basis using a standardized questionnaire within 24 h of delivery (or later in case a mother experienced some complications). In addition, women were asked to bring with their antenatal care card for information verification regarding pregnancy history. A verbal consent was sought from all mothers prior to the interview. Finally, all data was entered into the database at the medical birth registry.

The following information was collected from the mothers: Details on mother’s and father’s socio-demographic factors including occupation, education, age, marital status, alcohol and smoking during pregnancy, body mass index variables (pregnancy and the end of pregnancy). Details on obstetric characteristics as well as medical complications included pre-pregnancy conditions, mother’s conditions during pregnancy and after delivery. Information on pregnancy outcome include child status, still birth, birth weight and gestational age. The duration of hospital stay was calculated as the number of days from admission to the discharge date.

### Diagnosis of abruptio placentae

Diagnosis of abruptio placentae was based on routine clinical examination and ultrasound performed by the attending physician. The diagnosis was based on evidence of retroplacental clot accompanied by features clinical features such as vaginal bleeding, abdominal pain, abdominal tenderness, hypertonic uterus and hard abdomen with the fundal height greater than the gestational age accompanied.

#### Definition of outcome measures

*Postpartum haemorrhage* was defined as loss of blood estimated to be more than 500 mls from the genital tract, within 24 h of delivery. *Acute renal failure* was defined as an abrupt loss of kidney function resulting in the retention of urea and other nitrogenous waste products and in the dysregulation of extracellular volume and electrolytes.

*Preterm birth* was defined as a birth occurring at less than 37 completed weeks of gestation. *Perinatal death* was defined as fetal death that occurred inutero after a gestation age of 28 weeks or more (stillbirth), plus infant death that occurred at less than 7 days of age post delivery (early neonatal death). *Low birth weight infant* was defined as an infant having birth weight less than 2500 g. *Fetal distress* was defined as a compromised condition of the fetus, discovered during labor or intrapartum period characterized by a markedly abnormal heart rate or rhythm. *Apgar score* was defined as a measure of the physical condition of a newborn infant. It is obtained by adding points (2, 1, or 0) for heart rate, respiratory effort, muscle tone, response to stimulation, and skin coloration; a score of ten represents the best possible condition. *Pathology of lower genital tract* was defined as lower genital tracts lesions presenting with vaginal bleeding or spotting. *Abdominal trauma* was defined as blunt abdominal injury.

This study was approved by the Kilimanjaro Christian Medical University College Research Ethics Committee. The informed consent was obtained from all women prior the interview after they were fully explained the aim of the medical birth registry project. As for this study, informed consent was not requested as we used data from already approved project. Furthermore, confidentiality and privacy was assured according to the birth registry protocol.

### Statistical analysis

Data analysis was performed using Statistical package for Social Science (SPSS) program (SPSS Inc. Chicago, III) version 17.0. Maternal age was normally distributed, thus the difference in maternal age was assessed using student-*t* test. The differences involving categorical variable were assessed by chi-square test. Odds ratios (ORs) with 95 % confidence intervals (CIs) for the risk factors associated with abruptio placentae and maternal-foetal outcome were estimated in a multivariable logistic regression. A *P*-value of less than 0.05 (2-tailed) was considered to be statistically significant.

A variable was considered as a possible confounder if its inclusion in the model changed odds ratio of the independent variable of interest by at least 10 %. We considered the following variables as possible confounders in our analysis: maternal age, marital status, parity, alcohol use during pregnancy and area of residence.

## Results

### Demographic and obstetric characteristics of the study participants

A total of 39,993 deliveries were analysed. The incidence of abruptio placentae during the study period was 0.3 % (112/39,993). Demographic and obstetric characteristics of the participants are described in Table 1. Women with abruptio placentae had significantly higher mean age as compared to women without abruptio placentae, (29.1(SD = 5.4) vs.27.5 (SD = 6.0); *P* = 0.004, respectively). Married women and those who reported residing in rural areas were all at increased risk for abruptio placentae. Furthermore, the proportion of abruption increased with increasing education level and parity, although an association with maternal smoking and alcohol consumption during pregnancy was not significant.

**Table 1:** Demographic and obstetric characteristics of the study participants

Maternal characteristics	Placental abruption (*N* = 112; %)	No placental abruption (*n* =39,881; %)	*P* value*
Maternal age^a^	29.1 (5.4)	27.5 (6.0)	0.004
Maternal age (years)			0.02
<20	12 (10.7)	3528 ((8.8)	
20–34	80 (71.4)	30,782 (77.3)	
≥35	20 (17.9)	5571 (13.9)	
Marital status			0.03
Married	107 (95.5)	35,549 (89.1)	
Single	5 (0.5)	4332 (10.9)	
Mother’s area of residence			
Rural	61 (54.5)	16,995 (42.6)	0.01
Urban	51 (45.5)	22,886 (57.4)	
Smoking in current pregnancy			1.00
Yes	0 (0)	44 (0.1)	
No	112 (100)	39,837 (99.9)	
Alcohol use during pregnancy			0.12
Yes	44 (38.4)	12,679 (31.8)	
No	68 (60.7)	27,202 (68.2)	
Mother’s educational (years)			0.001
<12	87 (77.7)	25,081 (62.9)	
≥12	25 (22.3)	14,800 (37.1)	
Antenatal care (ANC)			0.54
Yes	111 (99.1)	39,607 (99.3)	
No	1 (0.9)	274 (0.7)	
Gravidity			<0.001
1	22 (19.6)	12,105 (30.4)	
2–4	67 (59.8)	24,618 (61.7)	
≥5	23 (20.6)	3158 (7.9)	
Parity			<0.001
0	24 (21.4)	13,056 (32.7)	
1–4	76 (67.9)	25,777 (64.6)	
≥5	12 (10.7)	1048 (2.6)	

^a^mean and standard deviation

*P value calculated from chi square

### Risk factors for abruptio placentae

The results from multivariate logistic model for risk factors associated with abruptio placentae are summarized in Table 2. The risk factors of abruptio placentae in this population were chronic hypertension (OR 4.1; 95 % CI 1.3–12.8), preeclampsia/eclampsia (OR 2.1; 95 % CI 1.1–4.1), prior caesarean section delivery (OR 1.3; 95 % CI 1.2–4.2), previous abruptio placentae (OR 2.3; 95 % CI 1.8–3.4). Similarly, women complicated by abruption placentae were more likely to have less than 4 antenatal care visits (OR 2.1, 95 % CI 1.2–2.5), multigravida (OR 2.1; 95 % CI 1.2–3.5) and high parity (OR 1.4; 95 % CI 1.2–8.6). Other known risk factors for abruptio placentae such as multiple gestations and male infant gender were not significantly associated with abruptio placentae in this population.

**Table 2:** Risk factors associated with placental abruption

Risk factors	Placental abruption (N = 112; 0.3 %)	No abruption (N =39,881; 99.7 %)	Unadjusted OR (95 % CI)	Adjusted OR (95 % CI)^a^
Chronic hypertension			4.5 (1.6–13.2)	4.1 (1.3–12.8)
Yes	3 (1.1)	269 (98.9)		
No	109 (0.3)	39,612 (99.7)		
Preeclampsia/eclampsia^b^			2.5 (1.6–5.2)	2.1 (1.1–4.1)
Yes	9 (0.6)	1622 (99.4)		
No	103 (0.3)	38,259 (99.7)		
Multiple gestations				
Yes	106 (0.28)	37,664 (99.7)	1.0 (0.5–2.3)	0.8 (0.3–1.9)
No	6 (0.27)	2217(99.7)		
Previous caesarean -section			1.5 (1.2–4.8)	1.3 (1.2–4.2)
Yes	22 (0.3)	6947 (99.7)		
No	90 (0.12)	32,934 (99.8)		
Previous abruptio placentae^c^			3.8 (2.3–6.4)	2.3 (1.8–3.4)
Yes	49 (0.7)	6658 (99.3)		
No	63 (0.19)	33,223 (99.8)		
Antenatal care visits			1.6 (1.2–2.6)	1.3 (1.1–2.4)
<4	53 (0.4)	13,315 (99.6)		
≥4	59 (0.2)	26,566 (99.8)		
Gravidity			2.2 (1.4–3.6)	2.1 (1.2–3.5)
Multigravida	90 (0.3)	27,766 (99.7)		
Primigravida	22 (0.18)	12,115 (99.8)		
Parity			1.7 (1.4–10.5)	1.4 (1.2–8.6)
≥2	65 (0.36)	17,836 (99.6)		
<2	47 (0.2)	22,045 (99.8)		
Sex of the baby				
Male	62 (0.3)	20,580 (99.7)	1.2 (0.9–1.7)	1.03(0.7–1.5)
Female	50 (0.25)	19,301 (99.75)		

^a^Adjusted for maternal age, maternal education level, marital status, area of residence

^b^Adjusted for previous abruptio placentae and chronic hypertension

^c^Adjusted for previous caesarean section delivery

### Maternal outcomes associated with abruptio placentae

Findings from logistic regression model for maternal outcomes associated with abruptio placentae are illustrated in Table 3. Compared with women without abruption, those with abruptio placentae had increased risk of antepartum haemorrhage (OR 11.5; 95 % CI 6.3–21.2), postpartum haemorrhage (OR 17.9; 95 % CI 8.8–36.4), caesarean section delivery (OR 5.6; 95 % CI 3.6–8.8) and need for blood transfusions (OR 9.6; 95 % CI 6.5–14.1). Similarly, women with abruptio placentae were more likely to have prolonged hospital stay (OR 3.5; 95 % CI 1.4–8.6) compared with those who had no abruptio placentae. Furthermore, abruptio placentae increased women’s likelihood of having been referred during labour (OR 3.8, 95 % CI 1.5–9.2). Our data also show that, women whose pregnancies were complicated by abruptio placentae had increased risks of altered liver function (OR 5.3, 95 % CI 1.3–21.6) and (OR 1.6; 95 % CI 1.5–1.8). There were four maternal deaths which were related to postpartum haemorrhage, corresponding to case fatality rate of 3.6 % (6/112). Moreover, patients with abruptio placentae had increased odds for induced labour as well as being transferred to intensive care unit, but the association did not reach statistical significance.

**Table 3:** Multiple logistic regression of maternal outcomes associated with abruptio placentae

Maternal outcomes	Abruption placental (*n* = 112)	No abruption (*n* =39,881)	Unadjusted OR (95 % CI)	Adjusted OR (95 % CI)^a^
Antepartum haemorrhage				
Yes	13(3.0)	420 (97.0)	11.9 (6.5–21.8)	11.5 (6.3–21.2)
No	99 (0.3)	39,461 (99.7)	1.0	1.0
Postpartum haemorrhage				
Yes	10 (7.6)	145 (92.4)	25.6 (12.4–52.8)	17.9 (8.8–36.4)
No	100 (0.3)	39,736 (99.7)	1.0	1.0
Model of delivery				
Caesarean delivery	85 (0.7)	12,914 (99.3)	6.6 (4.3–10.1)	5.6 (3.6–8.8)
Vaginal delivery	27 (0.1)	26,967 (99.9)	1.0	1.0
Acute renal failure				
Yes	1 (0.6)	153 (99.4)	2.4 (0.6–16.3)	2.2 (0.3–15.5)
No	111 (0.3)	39,728 (99.7)	1.0	1.0
Need for blood transfusion				
Yes	43 (1.9)	2248 (98.1)	10.1 (6.9–14.8)	9.6 (6.5–14.1)
No	69 (0.2)	37,633 (99.7)	1.0	1.0
Duration of hospitalization				
>4 days	18 (1.3)	1415 (98.7)	5.2 (2.3–15.4)	3.5 (1.8–9.6)
≤4 days	94 (0.2)	38,466(99.7)	1.0	1.0
Referred for delivery				
Yes	46 (0.5)	8841 (99.5)	2.3 (1.6–9.5)	2.1 (1.5–8.2)
No	66 (0.2)	31,040 (99.8)	1.0	1.0
Altered liver function test				
	2 (1.5)	128 (98.5)	5.6 (1.4–22.9)	5.3 (1.3–21.6)
	110 (0.3)	39,753 (99.7)	1.0	1.0
Admission to NICU				
Yes	23 (0.4)	5667 (99.6)	1.6 (1.2–2.5)	1.5 (0.9–2.4)
No	89 (0.3)	34,214 (99.7)	1.0	1.0

Multiple logistic regression with back ward elimination of maternal outcomes associated with placental abruption

^a^Adjusted for preeclampsia/eclampsia, chronic hypertension

### Fetal outcomes associated with abruptio placentae

The foetal outcomes associated with abruptio placentae have been summarized in Table 4. After adjustment for confounding variables, infants born to women with abruptio placentae had increased risk of low birth weight (OR 5.9; 95 % CI 3.9–8.7), perinatal death (OR 17.6; 95 % CI 11.3-27-3), early neonatal death (OR 5.3; 95 % CI: 3.8–17.9) and stillbirth (OR 23.7; 15.6–35.9) especially fresh stillbirth (OR 2.5; 95 % CI 1.5–4.2). In addition, infants born to women with abruptio placentae had increased risk of low Apgar scores (below 7) at 1st and 5th min with ORs of 5.3 (95 % CI 2.7–10.1) and 2.2 (95 % CI 0.9–4.8), respectively as compared to infants born to mothers without abruptio placentae. The association between abruptio placentae with preterm birth and fetal distress was not statistically significant. Overall, abruptio placentae contributed to perinatal mortality rate of 40 per 1000 births (74/1826).

**Table 4:** Multiple logistic regression of foetal outcomes associated with abruptio placentae

Fetal outcomes	Abruption placenta	Non abruption	Unadjusted OR (95 % CI)	Adjusted OR (95 % CI)^a^	*P* value
Low birth weight					
Yes	56 (1.1)	5269 (98.9)	6.9 (4.7–10.1)	5.9 (3.9–8.7)	<0.001
No	56 (0.2)	34,612(99.8)	1.0	1.0	
Perinatal death					
Yes	63 (3.5)	1762(96.5)	28.9 (19.8–42.1)	17.6 (11.3–27.3)	<0.001
No	49 (0.1)	38,119(99.9)	1.0	1.0	
Early neonatal death					
Yes	5 (1.5)	315 (98.5)	5.5 (2.1–15.2)	4.3 (1.8–9.9)	0.001
No	107 (0..3)	39,566(99.8)	1.0	1.0	
Stillbirth^b^					
Yes	58 (3.8)	1473 (96.2)	28.0 (19.3–40.7)	23.7 (15.6–35.9)	<0.001
No	54 (0.1)	38,408(99.9)	1.0	1.0	
Stillborn condition^c^					
Fresh	37 (7.2)	475 (92.8)	4.6 (2.2–9.7)	2.5 (1.5–4.2)	<0.001
Macerated	9 (1.9)	534 (98.3)	1.0	1.0	
Preterm birth					
Yes	30 (0.2)	4473 (99.8)	2.9 (1.1–7.4)	0.4 (0.1–7.1)	0.97
No	82 (0.3)	35,408(99.7)	1.0	1.0	
Fetal distress					
Yes	22 (0.3)	6947 (99.7)	1.2 (0.5–3.6)	0.8 (0.2–3.1)	0.69
No	90 (0.1)	32,934 (99.9)	1.0	1.0	
Apgar score at 1 min					
<7	41 (1.1)	3810 (98.9)	5.4 (3.6–12.3)	5.3(2.7–10.1)	<0.001
≥7	71 (0.2)	36,071(99.8)	1.0		1.0
Apgar score at 5 min					
<7	18 (0.7)	2292 (97.1)	2.9 (1.2–5.3)	2.2 (0.9–4.8)	0.054
≥7	94 (0.2)	37,589 (99.9)	1.0	1.0	

Multiple logistic regression with back ward elimination of perinatal outcomes associated with placental abruption

^a^Adjusted for parity and antenatal care visits chronic hypertension and preeclampsia

^b^Adjusted for stillborn condition3

^c^does not add to to tota because of missing values

## Discussion

### Main findings

In this study, we found that the incidence of abruptio placentae was 0.3 %. The risk factors for abruptio placentae were chronic hypertension, preeclampsia/eclampsia, previous caesarean delivery or abruptio placentae, poor attendance to antenatal care and high parity. Patients with abruptio placentae had increased risk of adverse maternal and foetal outcomes. Post-partum haemorrhage was an important predictor maternal deaths.

### Comparison with previous studies

The incidence of abruptio placentae in our study corresponds with incidence of 0.34 % which has been reported previously [18]. This figure also falls within the range of 0.3–2 %, reported by others [2, 3, 8, 13], but it is lower than what has been reported previously in Uganda and Israel [22, 23]. The difference in incidence could partly be explained by the differences in the studied populations, study designs and diagnostic criteria.

Previous studies reported the association between increased risk of abruptio placentae with chronic hypertension [19, 22, 24–26] and preeclampsia/eclampsia [15, 21, 25, 27–29]. Our results are in general agreement with previous reports, but they are contrast with that was reported by Wandabwa and colleagues [22], who observed that neither severity of blood pressure nor proteinuria were predictive factors of abruptio placentae. The possible reason for difference in findings could be explained by differences in nature of the studied populations and prevalence of these risk factors.

The second important finding of our study pertains to the association between previous caesarean delivery and increased risk of abruptio placentae. The risk of abruptio placentae increased by nearly 2-fold for women with prior caesarean section delivery. Similar observation was reported elsewhere [18–20, 30], but in our study was lower than the seven and ten-fold reported by Wandabwa et al. and Nayama et al. [22, 31]. The increased risk of abruption among women with prior caesarean delivery may imply that these women had ruptured scar from the previous caesarean. The differences in risk of abruption placentae between our study and those of others may be explained by the differences in pre-existing conditions which are associated with abruptio placentae between the studied populations.

Our study confirmed that abruptio placentae in a previous pregnancy is an important risk factor for abruptio placentae in subsequent pregnancies. This observation is in agreement with previous report [32]. An association between prior abruptio placentae and its recurrence in the subsequent pregnancies was demonstrated by Rasmussen and colleagues in a Norwegian cohort [33] were they reported a 5-fold increase in relative recurrence risk of abruptio placentae. This indicates that more persistent risk factors such as preeclampsia and chronic hypertension may play the role.

In the present study, the occurrence of abruptio placentae was higher in women with high parity and multigravid as compared to nulliparous or primigravid women. These results are in line with many previous studies [24, 25, 34–38], but was in contrast with findings of Sanchez and colleagues [21] who observed that neither of these factors were associated with abruptio placentae.

Previous studies have reported that both maternal cigarette smoking [4, 5] and alcohol consumption during pregnancy [27, 28, 39] are associated with abruptio placentae. In contrast to our study, these variables were not associated with abruptio placentae. The proportion of those who smoked was too low to assess the influence of smoking on abruptio placentae. Furthermore, lack of association between alcohol consumption and abruption placentae may in part be explained by the variation in amount, type and mode of alcohol used by participants between studies.

The occurrence of abruptio placentae has been associated with an increased risk of maternal and foetal morbidity and mortality in previous studies [1, 3, 6, 22]. In line with our study, women with abruptio placentae had 12-fold higher risk of postpartum haemorrhage as compared to women in the comparison group. Similarly, Sarwar and colleagues in Pakistan [10], reported that 22.2 % of the abruptio placentae patients experienced severe postpartum haemorrhage as a result of uterine atony, coagulation failure, or puerperal sepsis. However, it is worth noting that the diagnosis of abruptio placentae is subject to misclassification and is recorded with knowledge of the outcome of pregnancy. Therefore, pregnancy with severe haemorrhage may be more likely to be recorded as abruptio placentae if the baby dies. This in part could be a possible explanation for increased risk of postpartum haemorrhage attributed to abruptio placentae in our study.

The observed maternal death rate related to abruptio placentae in our study is above the maximum acceptable rate of 1 % [7], but the case fatality rate is lower than the 4.7 % that was reported by Seema et al. [6].

In our study, women with pregnancies complicated by abruptio placentae had six-fold risk of delivering an infant with low birth weight as compared to women in the control group. Similar finding was reported in Uganda [22]. The high risk of low birth weight delivery among women with abruptio placentae may be explained by the effect of preterm birth due to premature termination of pregnancy performed by clinicians due to severity of abruptio placentae or effect of intrauterine foetal growth retardation. The risks of stillbirth, early neonatal death and perinatal death were also higher among women with abruptio placentae as compared to women without abruptio. These results are in congruent with previous studies [1, 6, 9, 13, 21, 23]. It has been previously reported that the magnitude of stillbirth is dependent on the degree of separation of placentae, particularly when it exceeds 50 % [12]. This could be the case for some women in the present study, which in turn could be the reason for the observed high risk of perinatal death and its components. However, the observed excess risk in perinatal death may also likely to be caused by prematurity and intrauterine foetal growth restriction. It is worth noting that the perinatal mortality rate observed in our study is higher than 25.6 per 1000 deliveries reported elsewhere [6].

Previous investigators reported an increased risk of preterm birth among women with abruptio placentae [9]. However, this was not the case in our study. The association between abruptio placentae and preterm birth disappeared after adjustment for covariates in the model. Furthermore, patients with abruptio placentae were more likely to deliver a baby with low Apgar score at 1^st^ and 5^th^ minutes respectively. The high risk of low Apgar score may be due to prematurity, while on the other hand it may be an indication of lack equipment/supplies and lifesaving skills of new-borns who are born preterm/low birth weight in the study setting.

### Strengths and limitations of the study

The strength of this study is that we used maternally linked data which containing detailed information on paternal and maternal social demographic, mother’s information before and during pregnancy, labour and after delivery as well as new-born status. This information allowed us to examine the reproductive history of all women in the cohort over a 10 year period, thus providing an adequate sample size to examine with high precision for a relatively uncommon outcome. Secondly, the linked data also allowed us to measure and take into account the influence of important reproductive factors that confound the association between abruptio placentae and the studied outcomes thus avoiding overestimation of the reported risks. Third, we used data which was collected using a standardized questionnaire thus enhancing the quality of our data.

This study has some weakness. First, possibility of selection bias, since this was a hospital-based study, women with complicated pregnancies are more likely to be referred for delivery in the study setting. This may lead to overestimation of the reported risk and maternal fetal outcomes.

Secondly, diagnosis of abruptio placentae was based on an assessment made by the attending obstetrician. This subjective assessment may have resulted in some misclassification; especially for the severe and milder forms of abruptio placentae. Another weakness is the possibility of underreporting perinatal mortality. The medical birth registry at KCMC only captures information for mothers who stayed in the hospital up to 7 days after delivery. Therefore a ascertainment of early neonatal death may not be complete. Early neonatal deaths that may have occurred outside the hospital within 7 days of life for mothers who were discharged earlier than 7 days were not captured in the birth registry. This may have resulted to underreporting of true perinatal death.

## Conclusions

Our study confirmed that chronic hypertension, preeclampsia/eclampsia, previous caesarean delivery or abruptio placentae and high parity are independent risk factors for abruptio placentae. Abruptio placentae is associated with adverse maternal and fetal outcomes. Our results provide clinicians with import information to consider when counselling women during prenatal care about the risk factors for abruptio placentae, early identification of women at risk of abruptio placentae and develop individual follow up plan with specific intervention. Improved attendance to antenatal care and timely management of these factors may reduce the risk of abruptio placentae and associated adverse feto-maternal outcomes. Furthermore, better equipped obstetric and neonatal units to improve both maternal and perinatal outcomes in this setting are warranted.
